# ATTRN: Acoustic Information Encoder and Temperature Field Reconstruction Decoder Network for Boiler Temperature Field Reconstruction

**DOI:** 10.3390/s25082567

**Published:** 2025-04-18

**Authors:** Kunyu Wu, Keqi Ni, Liwei Chen, Hengyuan Xu, Junqiao Wang, Jingyi Zhou, Xinzhi Zhou

**Affiliations:** 1 Sichuan University Pittsburgh Institute, Sichuan University, Chengdu 610065, China; kun_2025@163.com (K.W.); nick.keqi@gmail.com (K.N.); xhy1270834639@gmail.com (H.X.); tlmsq@outlook.com (J.W.); zhoujy0628@163.com (J.Z.); 2 College of Design and Engineering, National University of Singapore, Singapore 117575, Singapore; childweichen@gmail.com; 3 College of Electronics and Information Engineering, Sichuan University, Chengdu 610065, China

**Keywords:** boiler furnace monitoring, acoustic temperature measurement, convolutional neural network, transformer, temperature field construction

## Abstract

Accurate and swift evaluation of the temperature distribution in boiler furnaces is essential for maximizing energy efficiency and ensuring operational safety. Traditional temperature field reconstruction algorithms, while effective, often suffer from accumulated errors, difficulty in solving ill-posed problems, low accuracy, and poor generalization. To overcome these limitations, a Temperature Field Reconstruction Network based on an acoustic information encoder (AIE) and a temperature field reconstruction decoder (TFRD) is proposed (ATTRN). This method directly utilizes acoustic measurement data for temperature field prediction, effectively balancing global semantic capture and local detail preservation. The proposed approach avoids complex traditional mathematical processing and empirical parameter selection, enhancing both accuracy and generalization. Simulation studies and engineering validations demonstrate the performance and industrial applicability of the proposed method.

## 1. Introduction

Boilers are the primary heat supply equipment in thermal power plants and play a crucial role in power generation [1,2]. The combustion process inside the furnace is complex and highly dynamic [3], and is considered one of the most intricate physical processes [4]. The stability of fuel combustion directly affects the safe operation of the entire system. Instabilities in the combustion process can lead to increased pollutant emissions, reduced combustion efficiency, and, in severe cases, even boiler explosions [5,6]. In practical engineering applications, the temperature field within the furnace serves as a direct indicator of the combustion state. Therefore, accurately reconstructing the furnace temperature field is essential to optimize combustion control, reduce pollutant emissions, and improve the operational reliability of boiler systems.

During the normal operation of a boiler, the furnace interior is typically subjected to a high-temperature environment. Due to the implementation of cooling measures, such as water-cooled walls in most boilers, the thermal distribution varies significantly across different regions, influenced by factors such as burner arrangement, furnace geometry, and water-cooling design. In certain designs, the central region exhibits relatively uniform temperatures, while areas farther from the flame or subjected to enhanced cooling by water-cooled tubes experience a sharp temperature drop. However, for different types of boilers, the temperature distribution within the furnace does not necessarily follow the pattern of a “uniform center with a decreasing periphery”, but rather varies according to the specific design and operational requirements of the boiler. To monitor the complex thermal environment inside the furnace, non-contact temperature measurement techniques have become a research focus [7,8]. These methods include laser thermometry, infrared thermometry, and acoustic thermometry [9,10], all of which allow temperature measurements in high-temperature and harsh environments without disturbing the target temperature distribution. Among these, acoustic thermometry has garnered widespread attention in recent years for furnace temperature monitoring and reconstruction due to its wide measurement range and low cost [11,12,13].

Acoustic-based temperature field reconstruction typically involves two key components: time-of-flight (ToF) measurement and temperature field reconstruction algorithms. ToF data are typically collected by various acoustic transceiver sensors and processed using time-domain, frequency-domain, or hybrid-domain methods [14]. Once reliable ToF data are obtained, the next critical step is to address the ill-posed problem through reconstruction algorithms to achieve a stable and reliable solution. The prevailing temperature field reconstruction algorithms can be categorized into non-iterative and iterative methods. Non-iterative methods primarily include the truncated singular value decomposition (TSVD) [15,16,17], Tikhonov regularization [18,19,20], and the least squares method (LSM) [21]. TSVD achieves a stable numerical solution by truncating singular values close to zero; however, as singular values continue to decrease, the stability of the solution deteriorates. The Tikhonov regularization method is well suited for solving inverse problems, but its reconstructed distributions tend to be overly smooth, leading to blurred edge details. LSM can only produce meaningful solutions when the number of grid points is smaller than the number of measurement paths, and its reconstruction results are often incomplete. Iterative methods mainly include the algebraic reconstruction technique (ART) [2,22,23], the simultaneous iterative reconstruction technique (SIRT) [24,25], the simultaneous algebraic reconstruction technique (SART) [26,27,28,29], and the Landweber iteration method [30,31]. ART is computationally simple and easy to implement, but it suffers from low accuracy and robustness of the reconstruction. SIRT requires a longer iteration time, whereas SART offers greater robustness compared to ART and achieves faster convergence. The Landweber method is the steepest descent iterative algorithm; however, it is prone to the issue of semi-convergence.

Existing temperature field reconstruction algorithms have made significant progress in reducing computational errors and improving reconstruction accuracy. However, cumulative errors and amplification effects persist, making it challenging for traditional methods to completely overcome their inherent limitations. Conventional algorithms primarily enhance numerical stability by optimizing the condition number of matrices, yet the final solution still exhibits unavoidable biases. Furthermore, block numerical methods introduce errors at each computational step, further constraining overall accuracy. The accuracy and robustness of temperature field reconstruction largely depend on parameter selection, such as the shape parameter of radial basis functions and the regularization parameter used to address ill-posed problems. These parameters typically require prior knowledge and extensive numerical experiments for tuning, thereby increasing the complexity of the algorithm and limiting its generalizability across different application scenarios.

In recent years, machine learning techniques have been widely applied to temperature field reconstruction to overcome the inherent limitations of traditional numerical methods. Among these, convolutional neural networks (CNNs) have emerged as one of the most commonly adopted architectures, with numerous reconstruction approaches built upon CNN-based frameworks. The temperature field residual correction network (TRCN) effectively mitigates solution bias in conventional reconstruction algorithms by employing residual learning and nonlinear error prediction mechanisms, thereby enhancing reconstruction accuracy [32]. Similarly, the fast response temperature field reconstruction network (FTRN) [33] leverages a CNN architecture for rapid temperature field estimation. Despite their popularity, CNNs face notable challenges when applied to time-of-flight (ToF) data, which inherently exhibit sequential characteristics. To adapt ToF sequences to the fixed input format expected by CNNs, the FTRN model introduces a preprocessing step that reshapes the sequence into a matrix. This transformation can introduce computational redundancy and may disrupt the original temporal structure of the data [34]. ToF data encode both temporal and spatial dependencies across multiple acoustic transmission paths. For example, in multipath acoustic propagation, certain paths may exhibit temporal correlations or thermoacoustic coupling, which require the modeling of long-range dependencies. However, conventional CNNs operate with fixed local receptive fields, making them inadequate for capturing such long-range contextual information—especially in long sequences—without significantly increasing network depth or employing dilated convolutions [35,36]. Moreover, CNNs generally assume structured two- or three-dimensional input formats; reshaping sequential ToF data to fit these formats may cause input–structure mismatches, resulting in feature redundancy or loss of essential sequential relationships [34]. These limitations are particularly critical in furnace environments, where complex thermoacoustic propagation demands the preservation of original physical dependencies across paths. In addition, standard convolutional kernels remain static during training, limiting their adaptability to dynamic spatiotemporal patterns. As demonstrated by dynamic filter networks, failure to adapt convolutional kernels to varying input distributions may result in suboptimal modeling of temporal variations in highly dynamic sequences [37].

Beyond CNN-based models, other machine learning techniques have also demonstrated effectiveness. Methods based on the Kernel Extreme Learning Machine (KELM) [10] have been employed for high-resolution temperature field reconstruction, utilizing kernel-based learning to minimize reconstruction errors and preserve fine-grained structural details. Furthermore, Transformer-based architectures have shown considerable promise. The deep transfer operator learning method [38], which incorporates Transformer blocks, has achieved impressive results in reconstructing temperature fields of lithium-ion battery packs. By integrating physics-informed neural networks and domain adaptation strategies, these models enhance reconstruction accuracy and generalizability while ensuring adherence to physical laws. Their ability to extract domain-invariant features enables robust and accurate reconstruction, even in previously unseen scenarios, thereby underscoring the potential of advanced neural architectures for complex temperature field modeling tasks.

However, the aforementioned traditional methods still exhibit the following limitations in global information modeling and edge temperature capture:

1. Cumulative errors and amplification effects can lead to distortions in reconstruction results under extreme conditions.
2. Parameter selection, such as regularization terms and basis function shape parameters, often relies on prior knowledge and extensive numerical experiments, resulting in limited generalization ability.
3. Given the dynamic and highly nonlinear characteristics of the furnace temperature field, balancing global semantic information and local detail representation remains a bottleneck that existing algorithms struggle to overcome.
4. Existing CNN-based models suffer from inherent limitations in capturing long-range dependencies across acoustic paths in ToF data due to their restricted local receptive fields. Although stacking multiple layers or employing dilated convolutions can extend the receptive field to some extent, these strategies significantly increase model complexity and still fall short of effectively modeling the global correlations among multiple propagation paths.
5. The inconsistency between the input acoustic ToF data and the output reconstructed temperature field distribution poses a bottleneck for machine learning models in data processing and feature distribution.

To address the limitations in existing temperature field reconstruction methods, including the difficulty of eliminating cumulative errors and amplification effects, high dependency on parameter selection, and insufficient capture of global information and edge temperature characteristics, this paper proposes a temperature reconstruction network (ATTRN) based on an acoustic information encoder (AIE) and a temperature field reconstruction decoder (TFRD). The acoustic information encoder adopts a Transformer architecture to model long-range dependencies, effectively encoding ToF data collected by acoustic transceivers to ensure a comprehensive representation of acoustic propagation information. Unlike RNNs or LSTMs, which are used primarily to predict the evolution of sequential data, our task aims to reconstruct image-like spatial temperature fields. RNN-based models process data sequentially and are limited in capturing global dependencies across multiple acoustic paths. Moreover, they are prone to vanishing gradients when dealing with long sequences. In contrast, the Transformer architecture handles the entire ToF sequence in parallel and uses self-attention to model global cross-path interactions effectively, which is essential for accurate spatial reconstruction. The temperature field reconstruction decoder utilizes a CNN structure, focusing on optimizing local details and enhancing spatial feature representation, thereby improving the accuracy of reconstructed temperature fields. This hybrid architecture explicitly matches the dual nature of the problem: the Transformer models the global structure and dependencies of ToF inputs, while the CNN preserves and enhances local gradient patterns essential for accurate edge temperature reconstruction. Moreover, by avoiding redundant matrix transformations and sequential bottlenecks, the Transformer achieves efficient information aggregation across distant time steps, while the CNN decoder ensures efficient local refinement with low computational overhead. Although some minor information loss may occur in convolutional preprocessing, the deep multi-scale structure of the CNN compensates for it through hierarchical feature recovery. By integrating these two architectures, the encoder precisely captures and characterizes acoustic propagation properties, while the decoder accurately reconstructs the temperature distribution. Their synergistic effect enables a more complete restoration of the temperature field. Ultimately, this structure enhances global feature extraction, improves the reconstruction accuracy of edge temperatures, and increases the robustness of the reconstruction process, allowing it to adapt to complex temperature field distributions and achieve more precise and stable temperature reconstruction.

The contributions of this paper are as follows:

1. The designed AIE module fully utilizes the global information of ToF data and works in conjunction with the TFRD module to achieve the transformation from ToF sequence input to temperature field distribution output.
2. The proposed ATTRN model, which integrates the AIE and TFRD architecture, significantly improves reconstruction accuracy and demonstrates excellent generalization ability, overcoming the limitations of traditional methods constrained by limited information availability, making it applicable to more complex scenarios.
3. The model enhances computational robustness and stability, making it suitable for various combustion conditions, thus facilitating efficient and stable temperature field reconstruction.
4. Further validation of the algorithm’s effectiveness was conducted using data from the boiler at Chongqing Qineng Power Plant, confirming the industrial practicality and applicability of the proposed approach.

In the implementation, the acoustic information encoder encodes the ToF data sequence and facilitates cross-path information interaction. A token encoding mechanism is adopted, where information blocks are first encoded and then processed by the Transformer to optimize feature representation. This structure allows the encoder to directly capture long-range dependencies between acoustic paths, without relying on recurrence or manual feature fusion, which improves both scalability and stability. The temperature field reconstruction decoder is responsible for feature upsampling, enhancing spatial details, and generating high-precision temperature field distributions. Its convolutional design is particularly effective for retaining spatial locality, capturing edge gradients, and restoring fine-scale structures critical for accurate temperature field reconstruction. By integrating machine learning with acoustic thermometry, the proposed method outperforms traditional LSM-based approaches and the CNN-based method in terms of reconstruction accuracy, computational stability, and robustness to temperature variations, offering a novel perspective for industrial furnace temperature field reconstruction.

## 2. Proposed Method

### 2.1. ATTRN Model

#### 2.1.1. Model Architecture Overview

**Figure 1:**
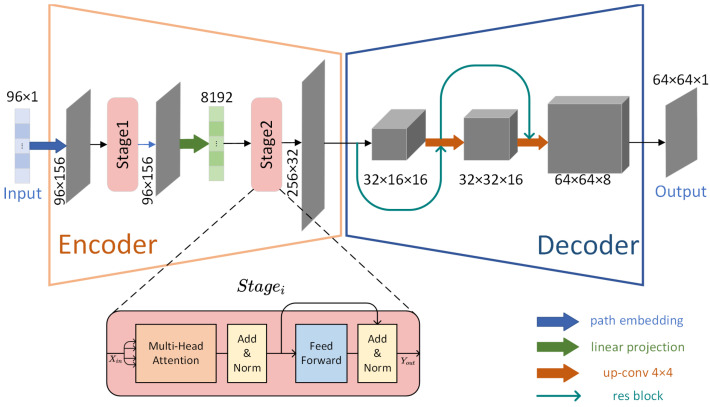
The model of ATTRN.

As illustrated in Figure 1, the ATTRN model employs a framework that integrates an acoustic information encoder (AIE) and a temperature field reconstruction decoder (TFRD), specifically designed to convert acoustic ToF sequence inputs into high-resolution 2D temperature field distributions. By leveraging specialized components in both the encoder and decoder, the model effectively transforms raw acoustic data into fine-grained temperature distribution.

The AIE is engineered to process acoustic ToF sequences—measurements that capture the time sound waves take to travel through a medium, influenced by temperature-dependent speed variations. It employs Transformer blocks to extract hierarchical features from these acoustic inputs, effectively distilling critical acoustic information that reflects the underlying temperature distribution. This process encodes the ToF sequences into semantically rich feature patches, preserving both local patterns (e.g., small-scale temperature variations) and global dependencies (e.g., broader thermal gradients). By focusing on acoustic information extraction, the encoder ensures that the encoded patches serve as a robust foundation for subsequent temperature field reconstruction.

The TFRD, leveraging the characteristics of CNNs, takes the encoded patches and reconstructs the temperature field with high fidelity. It integrates transpose convolutions, used for upsampling, to expand the spatial dimensions of the encoded features and residual blocks (resblocks) to refine and optimize spatial details. This design enables the TFRD to meticulously restore the temperature field, ensuring that the output—a high-quality 2D temperature field distribution—accurately represents the physical temperature distribution inferred from the acoustic ToF data. The emphasis on temperature reconstruction guarantees that the generated distribution captures both fine-grained details and overall thermal structures with precision.

#### 2.1.2. ATTRN Model Details

The AIE’s role is to transform input ToF sequences into feature representations of the entire temperature field. Its processing pipeline includes the following: path positional embedding, two layers of Transformer encoding the paths, projection of the encoded path sequences into patch sequences, and a final Transformer layer encoding the patch sequences.

This process begins with an input ToF sequence $x\in R^{N\times 1}$, which is projected through a linear layer to $x\in R^{N\times D_{1}}$, where *N* denotes the number of paths, and $D_{1}$ is the embedding dimension. Subsequently, the input sequence undergoes a linear transformation and is augmented with a learnable positional encoding $P\in R^{N\times D_{1}}$ to preserve temporal sequence information:

(1) $$E\left(x\right)=Linear_{e}\left(x\right)+P$$

where ${\mathrm{Linear}}_{e}$ is the projection layer, *e* denotes embedding, and *P* is the learnable positional encoder.

The resulting embedded sequence $E\left(x\right)\in R^{N\times D_{1}}$ is processed by the first Transformer encoder, which comprises two Transformer layers. This stage employs multi-head self-attention mechanisms and feed-forward networks to model complex inter-path dependencies, encoding them into embedded path tokens. Here, ${\mathrm{Enc}}_{1}$ is the stage-1 encoder and *T* represents the Transformer encoder:

(2) $$Enc_{1}\left(x\right)=T_{1}\left(E\left(x\right)\right)$$

where $T_{1}$ is the first-stage Transformer.

Next, the model converts path-based features (path tokens) into patch-based features. This is accomplished by flattening the encoded output, distributing it through a fully connected projection layer, and then reshaping the dimensions. This projection (*Proj*) generates a new set of tokens with an embedding dimension $D_{2}$ and a token count $T_{\exp}$, preparing for the spatial reconstruction of the temperature field:

(3) $$Proj\left(x\right)=Reshape(Linear\left(Flatten\left(Enc_{1}\left(x\right)\right)\right),\left(T_{exp},D_{2}\right))$$

where *Flatten* is the operation that eliminates the dimensional information, *Linear* is the fully connected projection layer, *Reshape* is the operation of reshaping to a specific dimension, $T_{exp}$ is the number of expanded tokens, and $D_{2}$ is the dimension of the expanded tokens.

Following projection, the expanded semantic patches are processed by a second Transformer encoder, consisting of one Transformer layer. This layer performs global, multi-level modeling of the patch tokens, refining their encoded information to improve the quality of the reconstruction temperature field. Accordingly, the final output of AIE can be formally represented as:

(4) $$AIE\left(x\right)=Enc_{2}\left(x\right)=T_{2}\left(Proj\left(x\right)\right)$$

where ${\mathrm{Enc}}_{2}$ is the stage-two encoder, and $T_{2}$ is the second-stage Transformer.

The TFRD’s function progressively decodes the encoded patch features into a 2D temperature field. This process occurs in two steps: first, spatial resolution is enhanced via transpose convolutions (upsampling); then, residual blocks further optimize the features, enhancing local details and minimizing artifacts. After two iterations of this process, a final convolutional layer (*FinalConv*) outputs the reconstructed temperature field—a 2D temperature distribution with dimensions $R^{1\times W_{\mathrm{out}}\times H_{\mathrm{out}}}$, where *W* is the width, and *H* is the height of the model’s output feature distribution. For any given input *x*, the output of TFRD can be formally expressed as:

(5) $$TFRD\left(x\right)=FinalConv(\psi_{2}\left(Up_{2}\left(\psi_{1}\left(Up_{1}\left(x\right)\right)\right)\right))$$

where *FinalConv* is the final convolution layer, $\psi_{1}$ is the series of resblocks after the first up convolution layer, $\psi_{2}$ is the series of resblocks after the second up convolution layer, ${\mathrm{Up}}_{1}$ is the first up convolution layer, ${\mathrm{Up}}_{2}$ is the second up convolution layer.

In summary, for a given input *x*, the forward propagation of the entire ATTRN model can be formally expressed as:

(6) $$ATTRN\left(x\right)=TFRD\left(AIE\left(x\right)\right)=FinalConv(\psi_{2}\left(Up_{2}\left(\psi_{1}\left(Up_{1}\left(Enc_{2}\left(Proj\left(Enc_{1}\left(E\left(x\right)\right)\right)\right)\right)\right)\right)\right))$$

### 2.2. Loss Function

The training objective of ATTRN is to enable the model to generate accurate and physically consistent temperature field distributions from ToF sequences. To achieve this, a hybrid loss function is employed during training to balance two critical reconstruction goals: reconstruction fidelity (ensuring the predicted temperature field closely matches the true temperature field) and smoothness (ensuring the reconstructed temperature field is continuous and aligns with real temperature distributions). This hybrid loss function combines mean squared error (MSE) loss and mean absolute error (MAE) loss, each weighted appropriately for backpropagation to optimize the model’s performance across these objectives. The specific rationale for selecting these loss functions is detailed below:

#### 2.2.1. MSE Loss

MSE Loss serves as the foundation for the model to accurately capture the position and magnitude of the temperature distribution. By penalizing the network with the average squared difference between the reconstructed and original temperature fields, it heightens the network’s sensitivity to regions with significant temperature disparities, reducing large errors and enhancing its ability to pinpoint the location and intensity of heat or cold sources. The MSE Loss is defined as follows:

(7) $$L_{\mathrm{MSE}}\left(p,q\right)=\frac{1}{n}\sum_{i=1}^{n}{\left(p_{i}-q_{i}\right)}^{2}$$

where *p* is the original temperature field distribution, *q* is the temperature field distribution predicted by ATTRN, *n* is the number of grid points in the temperature field distribution, $p_{i}$ and $q_{i}$ denote the temperatures at the *i*-th grid point in the original and predicted temperature fields, respectively.

#### 2.2.2. MAE Loss

Complementing MSE Loss, MAE Loss calculates absolute errors, linearly reflecting all discrepancies, unlike MSE Loss’s amplification of large temperature errors. This linear approach enables the correction of minor local differences, which is vital for improving the smoothness and realism of transitions between temperature zones in ATTRN’s predictions. Smoothness is critical for ensuring the physical plausibility of the temperature field, as real temperature distributions lack abrupt changes. The MAE Loss is expressed as:

(8) $$L_{\mathrm{MAE}}\left(p,q\right)=\frac{1}{n}\sum_{i=1}^{n}\left|p_{i}-q_{i}\right|$$

where *p*, *q*, *n*, $p_{i}$, and $q_{i}$ are as defined in the MSE Loss expression.

#### 2.2.3. Total Training Loss Function of ATTRN

**Figure 2:**
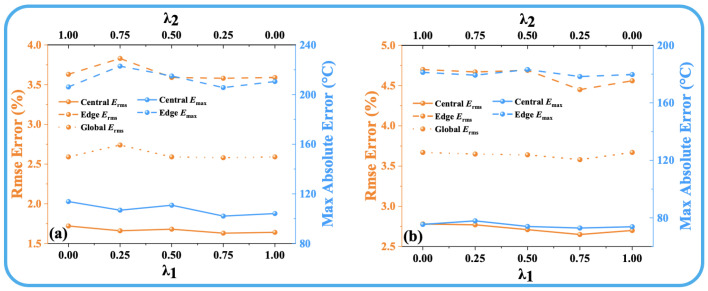
(**a**) Discrete fields. (**b**) Test-set error curves for different $\lambda_{1}$ and $\lambda_{2}$ in 54 paths.

**Figure 3:**
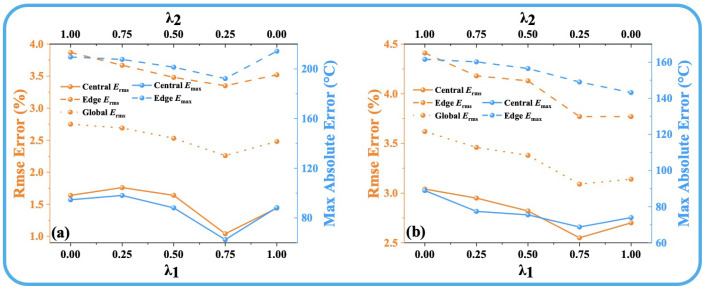
(**a**) Discrete fields. (**b**) Test-set error curves for different $\lambda_{1}$ and $\lambda_{2}$ in 96 paths.

The total training loss function of ATTRN is a linear combination of MSE Loss and MAE Loss, formulated as:

(9) $$L_{\mathrm{ATTRN}}=\lambda_{1}L_{\mathrm{MSE}}\left({Dist}_{target},{Dist}_{predicted}\right)+\lambda_{2}L_{\mathrm{MAE}}\left({Dist}_{target},{Dist}_{predicted}\right)$$

where $Dist_{target}$ is the target temperature field, and $Dist_{predicted}$ is the ATTRN output distribution. The parameters $\lambda_{1}$ and $\lambda_{2}$ are the weights assigned to the MSE Loss and MAE Loss, respectively, and their sum is constrained to 1. Figure 2 and Figure 3 illustrate the reconstruction errors (the details of the error are shown in Section 3.2) under different values of $\lambda_{1}$ and $\lambda_{2}$. The results clearly indicate that the best performance across all error metrics is achieved when $\lambda_{1}$ is set to 0.75 and $\lambda_{2}$ is set to 0.25. Therefore, this weighting configuration is adopted in the subsequent experiments to ensure optimal reconstruction accuracy.

## 3. Experiments and Results

### 3.1. Dataset Construction

**Figure 4:**
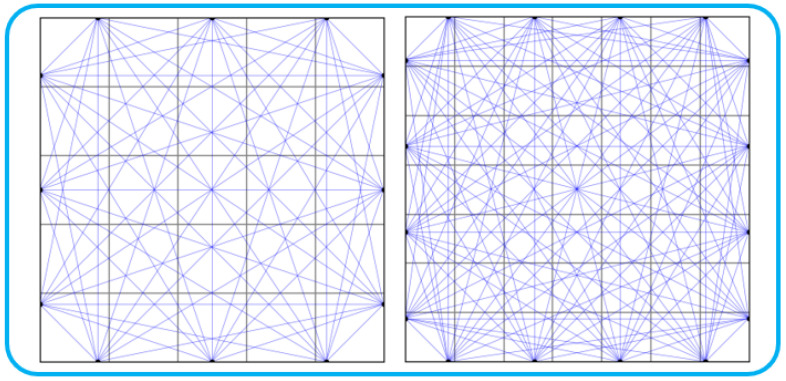
Sensor and path distributions for 54 paths (**left**) and 96 paths (**right**).

Function-based temperature field simulation methods are widely adopted in the literature [39,40,41]. The dataset used to train ATTRN and compare it with other methods is constructed based on the Acoustic Temperature Field Reconstruction Simulation Dataset (ATFRSD) from [32]. In this study, ATFRSD-96 and ATFRSD-54 are generated according to different sensor layouts and boundary conditions. Specifically, ATFRSD-96 consists of data collected from 16 boundary sensors, forming 96 propagation paths, while ATFRSD-54 comprises data from 12 boundary sensors, forming 54 propagation paths, as illustrated in Figure 4.

In practical engineering applications, furnace chambers typically incorporate water-cooled wall designs, leading to a rapid attenuation of the thermal gradient at the temperature field edges. To account for this characteristic, this study additionally constructs datasets that include the effects of water-cooled boundaries, named ATFRSD-96W and ATFRSD-54W, respectively. The scale and structure of these datasets remain consistent with their non-water-cooled counterparts. Each dataset comprises a total of 6400 samples, of which 5760 are allocated for training and 640 are reserved for testing, with all samples selected using stratified sampling. Each sample includes ToF measurement data along with the corresponding temperature field distribution, ensuring a comprehensive representation of temporal and spatial features to support model training and evaluation.

The generation of simulated temperature field data follows a controlled numerical modeling process. The initialization of each temperature field is given by:

(10) $$T\left(x,y\right)=T_{\mathrm{background}}\left(x,y\right)$$

where $T_{\mathrm{background}}\left(x,y\right)$ is randomly sampled within a predefined range. To introduce spatial variability, a random number of elliptical heat sources and cold sources are superimposed on the background temperature field. Each heat or cold source *k* contributes an additional Gaussian distribution term:

(11) $$T\left(x,y\right)\leftarrow T\left(x,y\right)\pm E_{k}e^{-\frac{{\left(x-A_{k}\right)}^{2}}{C_{k}}-\frac{{\left(y-B_{k}\right)}^{2}}{D_{k}}}$$

where the negative sign represents cold sources, determined by a specific probability range. The parameters $\left\{A_{k},B_{k},C_{k},D_{k},E_{k}\right\}$ are determined through random sampling to ensure diversity in position, spatial extent, and amplitude.

For datasets incorporating water-cooled walls, a boundary cooling mechanism is adopted to simulate the rapid heat dissipation process at the furnace edges in a discretized manner:

(12) $$T\left(x,y\right)\leftarrow T\left(x,y\right)+\sum_{\gamma \in \{L,R,T,B\}}\Delta T_{\gamma }\left(x,y\right)$$

where $\Delta T_{\gamma }\left(x,y\right)$ includes the cooling coefficient $\alpha_{\gamma }$, the wall temperature $T_{\mathrm{wall},\gamma }$, and the boundary width parameter $\omega_{\gamma }$. These adjustments ensure the formation of a steep thermal gradient near the boundary.

(13) $$c\left(x,y\right)=k\sqrt{T(x,y)}$$

where *k* is a constant with a value of 20.03. Therefore, the total ToF value for each path is obtained by accumulating the propagation time of all segments.

In each synthesized sample, the ToF is calculated along the propagation paths from boundary to boundary. A path from $\left(x_{1},y_{1}\right)$ to $\left(x_{2},y_{2}\right)$ is discretized into multiple segments, where the local acoustic velocity of each segment is determined by the following. The governing equations involved in the dataset generation process are detailed in Equations (10)–(13).

Table 1 and Table 2 summarize the primary randomization ranges of these parameters. By incorporating randomized background temperatures, multiple superimposed heat/cold sources, and water-cooled wall effects, the dataset ultimately comprises 6400 training samples, covering a wide range of temperature field configurations. Each sample consists of the following two components:

1. The final temperature field distribution has a resolution of 64 × 64.
2. The corresponding ToF vector (with a dimension of 54 or 96, depending on the sensor layout).

It is important to note that the parameter ranges are deliberately set to be relatively broad. This design choice aims to enhance the information extraction capability and generalization ability of the neural network. Since the dataset is generated based on simple exponential functions, there is a potential risk of overfitting—where the neural network may learn specific parameters of the heat sources rather than capturing the overall variations in the temperature distribution. By expanding the parameter range, we ensure that the shapes of heat sources within the temperature fields are not highly uniform, thereby forcing the encoder to extract local temperature variations more effectively rather than merely memorizing the parameters of the generating function. This strategy further improves the network’s performance on real-world data.

### 3.2. Experimental Indicators

To better assess the precision of the reconstruction, we introduce the following performance metrics [32]:

(14) $$E_{\max}=\max\left|TR_{i}-T_{i}\right|$$

(15) $$E_{\mathrm{rms}}=\frac{1}{T_{\mathrm{mean}}}\;\cdot \;\sqrt{\frac{\sum_{i=1}^{n}{\left(TR_{i}-T_{i}\right)}^{2}}{n}}\times 100\%$$

where $E_{\max}$ (unit: °C) represents the maximum absolute error in the measurement field, while $E_{\mathrm{rms}}$ denotes the root mean square error (RMSE) of the measurement field. The error calculation is conducted over a region containing *n* temperature sampling points, where n=64×64=4096. In this formulation, $T_{i}$ represents the temperature value of the *i*-th sampling point in the temperature field model, with i=1,2,…,n, while $TR_{i}$ represents the reconstructed temperature value at the same location after applying a given algorithm. $T_{\mathrm{mean}}$ is the mean temperature of the temperature field model. To compare the generalizability of different algorithms, the comparison involves evaluating the computational results of each algorithm on real datasets, using the mean error indicators $E_{\max}$ and $E_{\mathrm{rms}}$. Additionally, due to the potential loss of edge information in the LSM, we further introduce edge error, central error, and global error to provide a more comprehensive assessment of algorithm performance.

### 3.3. Robustness Evaluation of the ATTRN Framework

To assess the robustness of the ATTRN framework, we conducted experiments to analyze the impact of noise on ToF measurements. A detailed noise impact analysis indicates that the probability distribution of combustion noise within the furnace typically follows a Gaussian (GS) distribution. Based on the methods described in [42,43], we introduced additive Gaussian noise into the ToF data, with a mean value of 0 and standard deviations of 0.00005 s (low noise), 0.0001 s (medium noise), and 0.0002 s (high noise).

Subsequently, robustness tests were conducted under two different sensor configurations. In one configuration, 16 sensors were deployed along the boundary of the region, corresponding to 96 propagation paths. In the other configuration, 12 boundary sensors were used, corresponding to 54 propagation paths. Both sensor configurations included water-cooled walls to simulate the steep temperature gradient at the boundaries.

**Figure 5:**
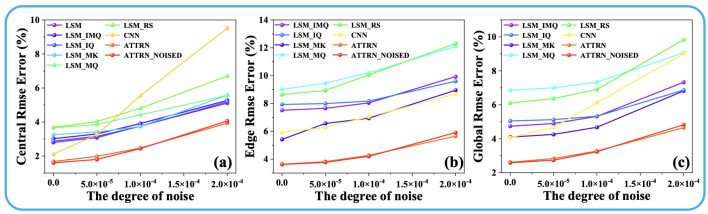
(**a**) Central, (**b**) edge, and (**c**) global error curves for different noise degrees in 54 paths.

**Figure 6:**
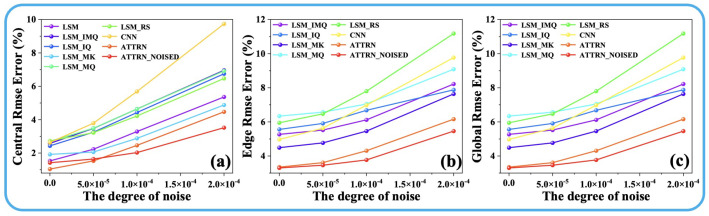
(**a**) Central, (**b**) edge, and (**c**) global error curves for different noise degrees in 96 paths.

As illustrated in Figure 5 and Figure 6, the experimental results demonstrate the performance of ATTRN under varying noise levels. In the case of 54 propagation paths (Figure 5), the central error of ATTRN increases from 1.7% to 3.9%, the edge error rises from 3.6% to 5.6%, and the global error increases from 2.6% to 4.6% as the noise level increases from 0 to 0.0002 s. This corresponds to an approximate error increase of 2.0%. In addition, the central error of the LSM-based approaches generally increases from 3.3% to 6.0%, with the LSM_RS rising from 3.7% to 6.9%. Regarding edge error, LSM_MK increases from 5.5% to 9.0%, LSM_IMQ and LSM_IQ increase to approximately 10.0%, while LSM_MQ and LSM_RS increase from around 9.0% to 12.5%. For global error, LSM_IQ rises from 4.1% to 6.8%, LSM_IMQ and LSM_IK from 4.9% to 7.2%, and LSM_MQ and LSM_RS from 6.5% to 9.5%. These results indicate that the LSM-based experiences an average increase in error of approximately 3–3.5% as noise increases to 0.0002 s. The CNN-based method shows a central error increase from 2.1% to 9.5%, edge error from 5.9% to 8.8%, and global error from 4.1% to 9.1%, reflecting an overall error increase of approximately 4–7.5%. For the 96-path configuration (Figure 6), the central error of ATTRN increases from 1.1% to 4.4%, edge error from 3.4% to 6.1%, and global error from 2.2% to 4.1%, corresponding to an increase of approximately 2.0–3.0% as noise increases. Meanwhile, the central error of LSM and LSM_MK increases from 1.8% to 5.2%, while the other four LSM-based methods increase from 2.7% to 6.9%. In terms of edge error, LSM_MK increases from 4.5% to 7.7%, LSM_IMQ and LSM_IQ increase to approximately 8.1%, LSM_MQ from around 6.0% to 9.1%, and LSM_RS from 6.0% to 11.1%. For global error, except LSM_IQ which increases from 4.8% to 6.8%, other LSM-based approaches show an average increase of approximately 3.5%. These results suggest that the LSM-based approaches experience an error increase of about 3.4–5.1% as the noise level reaches 0.0002 s. For the CNN-based method, the central error increases from 2.6% to 9.7%, edge error from 5.0% to 9.8%, and global error from 3.7% to 9.6%, corresponding to an overall error increase of approximately 4.8–6.9%.

In summary, the results presented in Figure 5 and Figure 6 indicate that the ATTRN framework consistently outperforms both traditional LSM-based approaches and CNN-based methods in temperature field reconstruction under various levels of noise interference. This advantage primarily stems from the synergistic interaction between AIE and TFRD. Firstly, the Transformer architecture within AIE effectively models long-range dependencies, enabling it to extract global features even in the presence of noise perturbations in ToF data. This capability mitigates the impact of noise on the overall data trend. Additionally, the CNN-based TFRD enhances spatial detail recovery through multi-scale feature extraction, allowing it to efficiently filter small-scale noise interference. Moreover, the end-to-end learning framework enables ATTRN to adaptively adjust feature weights, ensuring strong robustness across varying noise levels. Compared to traditional methods, the ATTRN framework, when trained under noiseless conditions, achieves a significant reduction in temperature reconstruction error by approximately 2% to 6% under noiseless, low-noise, medium-noise, and high-noise conditions. Furthermore, as illustrated in Figure 5 and Figure 6, training the ATTRN framework under low-noise conditions enhances its noise resistance, demonstrating the inherent robustness of neural networks. This finding underscores ATTRN’s superior capability in handling noisy ToF measurement data, further highlighting its enhanced robustness and reliability in practical applications.

### 3.4. Comparison Experiment

To comprehensively compare the performance of different algorithms, we conducted experiments under the ATTRN framework, incorporating multiple LSM-based approaches and CNN-based methods. The coefficient matrix in the LSM formulation is of full rank, and therefore, the optimization problem can be solved directly without the need for iterative methods. These methods were evaluated on the ATFRSD-54, ATFRSD-54w, ATFRSD-96, and ATFRSD-96W datasets to assess their effectiveness in temperature field reconstruction. The experiments considered two error metrics: $E_{\max}$ (°C) and $E_{\mathrm{rms}}$. The Table 3, Table 4, Table 5 and Table 6 present the quantitative performance metrics for eight algorithms. It is important to note that we did not separately compare the single-peak, double-peak, and high-low-peak temperature fields. Instead, we directly compared the average $E_{\max}$ and $E_{\mathrm{rms}}$ values across all three cases. This approach was adopted because the performance trends of traditional LSM-based approaches, CNN-based method, and ATTRN were consistent across these three temperature field distributions. In other words, each method exhibited a similar pattern of error metrics across different temperature fields. Therefore, using the average values for comparison not only improved evaluation efficiency but also ensured statistical robustness without compromising the validity of the conclusions. Experimental results indicate that compared to traditional methods, ATTRN achieves significant improvements in central region, edge region, and global error metrics. On the ATFRSD-96W test set, the global error of ATTRN is only 3.09%, significantly lower than LSM_RS (28.06%), LSM_MQ (22.64%), and CNN (20.93%). Furthermore, in the ATFRSD-54W test set, the edge error of ATTRN (4.78%) is notably lower than that of LSM_RS (14.09%), with a reduction of approximately 10%. This demonstrates that ATTRN achieves higher reconstruction accuracy in edge regions and is better suited to capturing the complex temperature gradient distribution within the furnace. Moreover, in test sets without water-cooled walls, ATTRN maintains an error of approximately 3% across central, edge, and global regions, which remains lower than the 3–12% error observed in LSM-based approaches and CNN-based method. In addition to average reconstruction accuracy, ATTRN also exhibits a remarkable reduction in $E_{\max}$, which is critical for ensuring the stability and safety of thermal field monitoring. In the ATFRSD-96W test set, ATTRN achieves the lowest central $E_{\max}$ of only 68.72 °C, while all LSM-based approaches report significantly higher values ranging from 751.04 °C to 1082.34 °C, and the CNN-based method even reaches a peak error of 1227.39 °C. This trend is similarly evident in edge regions, where ATTRN’s $E_{\max}$ is merely 149.02 °C, in stark contrast to the 1170.17 °C of the CNN model and the extreme value of 1689.69 °C observed in LSM_RS. A similar advantage is observed in test sets without water-cooled walls. In the ATFRSD-54 test set, ATTRN achieves a central $E_{\max}$ of only 104.47 °C, whereas LSM-based approaches range from 120.05 °C to 252.51 °C, and the CNN-based model reaches a $E_{\max}$ of 1324.83 °C. For edge regions, ATTRN once again attains the lowest $E_{\max}$ at 132.28 °C, which is significantly lower than the values observed in LSM methods (ranging from 280.93 °C to 702.44 °C) and the CNN-based method (1336.45 °C). Thus, ATTRN consistently outperforms other comparative methods, exhibiting higher reconstruction accuracy under different sensor layouts and boundary conditions. Notably, as the number of heat sources increases and temperature variations become more complex, ATTRN demonstrates superior adaptability. Evaluations based on real-world datasets further validate this advantage, confirming the robustness and effectiveness of ATTRN in practical applications. Therefore, it can be concluded that ATTRN provides a comprehensive and superior solution for temperature field reconstruction. Compared to CNN-based methods, traditional LSM and its variants, ATTRN, maintain high accuracy, primarily due to its advanced AIE-TFRD architecture, which we designed. This structure possesses powerful global information-encoding capabilities and efficiently maps acoustic information to temperature field distributions. These findings further highlight the robustness and reliability of ATTRN in handling complex temperature distributions, enhancing its feasibility for real-world applications. Additionally, the test datasets were generated using previously proposed methods, ensuring consistency in evaluation. The superior performance of ATTRN over other methods suggests its capability to handle more challenging conditions, such as a higher number of heat sources and more complex temperature variations.

In summary, the extensive experimental results presented in Table 3, Table 4, Table 5 and Table 6 demonstrate that the proposed ATTRN outperforms traditional algorithms and the CNN-based method in terms of the accuracy of reconstruction. ATTRN not only improves reconstruction precision but also effectively reduces reconstruction bias compared to baseline algorithms, thereby achieving a lower global error, central error, and edge error. In addition, the number of heat sources significantly affects the accuracy of the prediction. Traditional methods perform well when the number of heat sources ranges from 1 to 3; however, as the number of heat sources exceeds 3, their accuracy drops sharply, leading to a rapid increase in error. This decline is particularly pronounced in the edge regions, where the accuracy of the reconstruction degrades the most. In contrast, ATTRN maintains consistently high performance even in scenarios with multiple heat sources, highlighting its strong generalization capability and robustness in complex environments. Table 7 and Table 8 provide the parameters of the ATTRN model for the 54-path and 96-path configurations, respectively.

### 3.5. Engineering Validation and Analysis

All engineering experiments conducted in this study follow the same test scenarios and data conditions as described in [32]. The experimental subject of this study is a 330 MW subcritical natural circulation drum boiler at Chongqing Qineng Power Plant. The burner configuration, operating modes, evaporation rate, and typical working parameters of this boiler are detailed in Table 9. To acquire furnace temperature field data, a comprehensive acoustic measurement system was employed. This system, manufactured by China Dongfang Boiler Control Co., Ltd., Zigong, China, consists of key components, including acoustic transceivers, preamplifiers, a signal processing controller, an Ethernet switch, and a host computer. The acoustic transceivers serve as the primary components for transmitting and receiving acoustic signals. The transmission unit utilizes a pneumatic sound source, where an electromagnetic valve regulates compressed air to generate acoustic signals. The transmission frequency is set at 3 kHz, with a signal intensity exceeding 130 dB. To withstand high-temperature environments, the receiving unit is equipped with an air-cooling mechanism, supporting a reception frequency range of 20 Hz–20 kHz, with a microphone sensitivity of approximately −26 dB. Additionally, the preamplifier is responsible for adjusting the gain of received acoustic signals, featuring an input impedance of 100 M ohm and a gain factor set to 0.5. The signal processing controller operates with a 12-bit synchronous sampling mode, using a sampling frequency of 20 kHz to ensure precise signal processing. The entire measurement system is connected to the host computer via a 100 M industrial Ethernet network, ensuring stable data transmission and real-time analysis.

**Figure 7:**
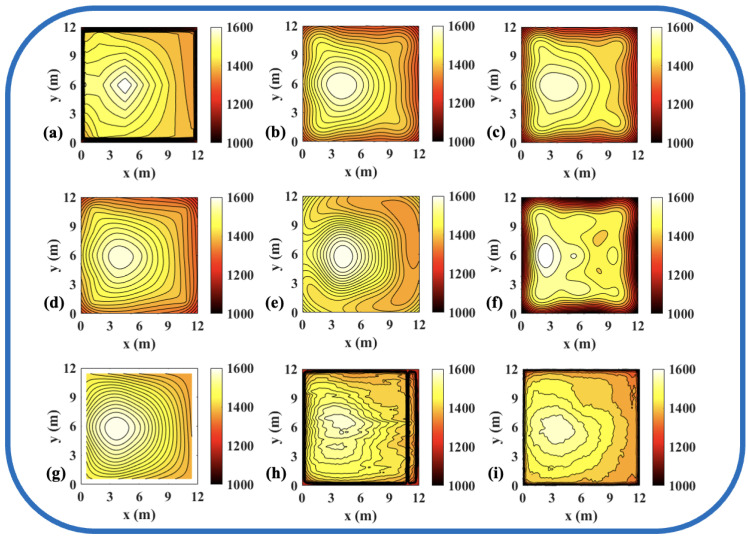
Reconstruction results of experimental single-peak temperature field in 54 paths. (**a**) Discrete field. (**b**) LSM_IMQ. (**c**) LSM_IQ. (**d**) LSM_MK. (**e**) LSM_MQ. (**f**) LSM_RS. (**g**) LSM. (**h**) CNN. (**i**) ATTRN.

**Figure 8:**
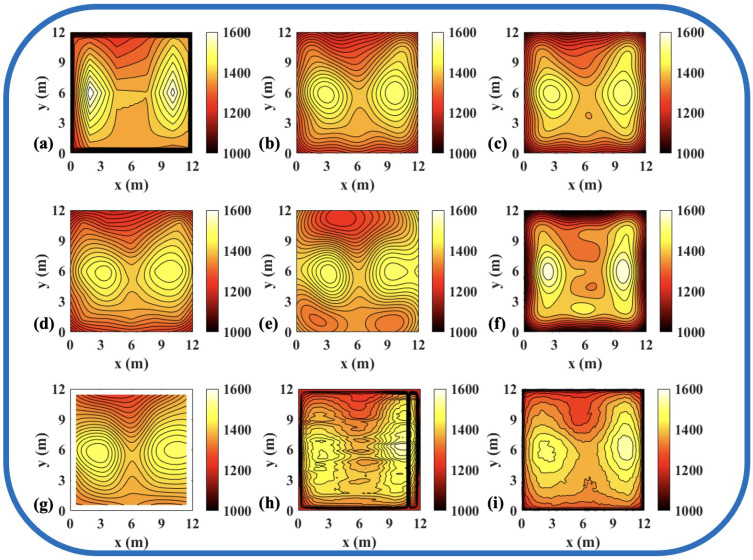
Reconstruction results of experimental double-peak temperature field in 54 paths. (**a**) Discrete field. (**b**) LSM_IMQ. (**c**) LSM_IQ. (**d**) LSM_MK. (**e**) LSM_MQ. (**f**) LSM_RS. (**g**) LSM. (**h**) CNN. (**i**) ATTRN.

**Figure 9:**
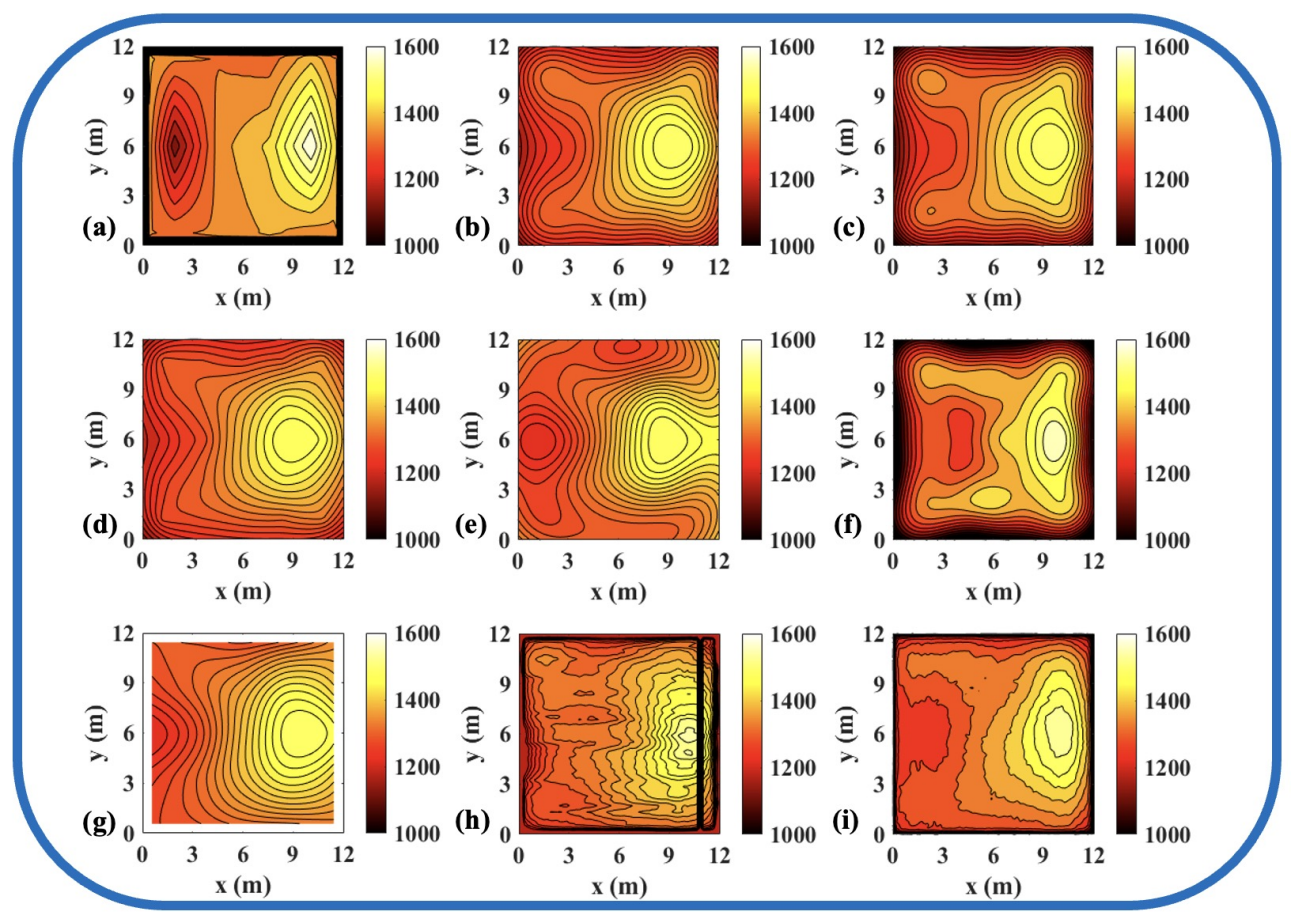
Reconstruction results of experimental high-low temperature field in 54 paths. (**a**) Discrete field. (**b**) LSM_IMQ. (**c**) LSM_IQ. (**d**) LSM_MK. (**e**) LSM_MQ. (**f**) LSM_RS. (**g**) LSM. (**h**) CNN. (**i**) ATTRN.

**Figure 10:**
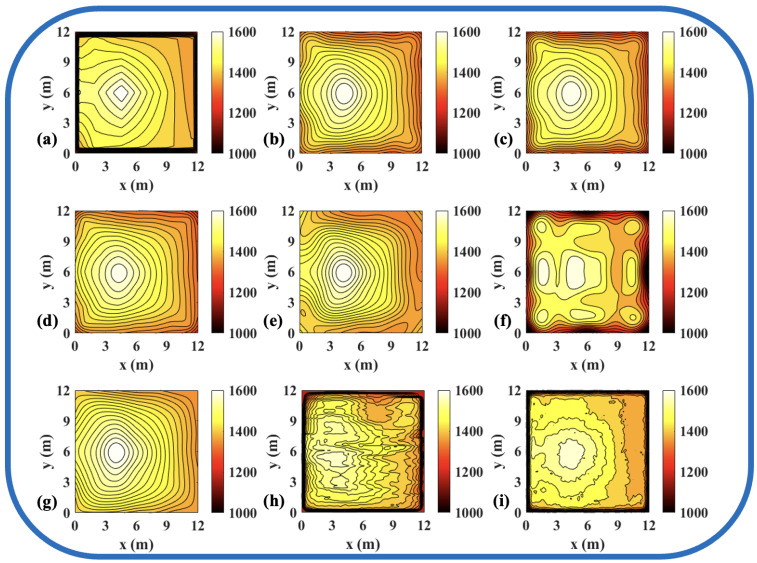
Reconstruction results of experimental single-peak temperature field in 96 paths. (**a**) Discrete field. (**b**) LSM_IMQ. (**c**) LSM_IQ. (**d**) LSM_MK. (**e**) LSM_MQ. (**f**) LSM_RS. (**g**) LSM. (**h**) CNN. (**i**) ATTRN.

**Figure 11:**
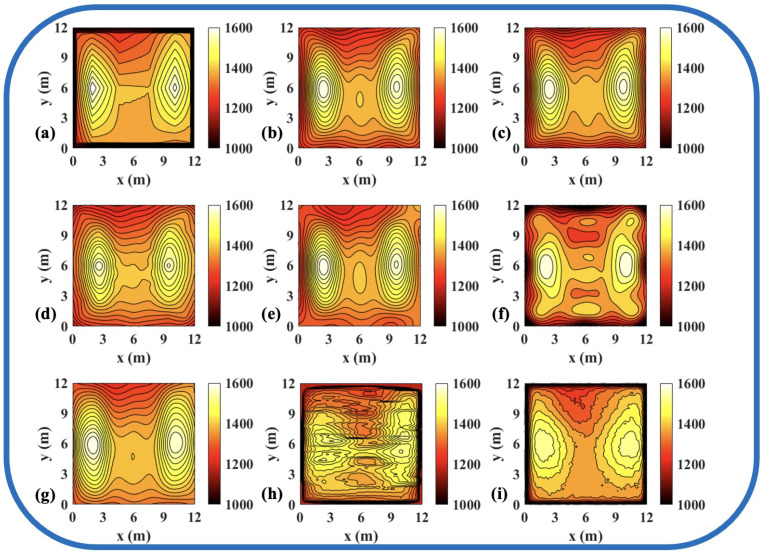
Reconstruction results of experimental double-peak temperature field in 96 paths. (**a**) Discrete field. (**b**) LSM_IMQ. (**c**) LSM_IQ. (**d**) LSM_MK. (**e**) LSM_MQ. (**f**) LSM_RS. (**g**) LSM. (**h**) CNN. (**i**) ATTRN.

**Figure 12:**
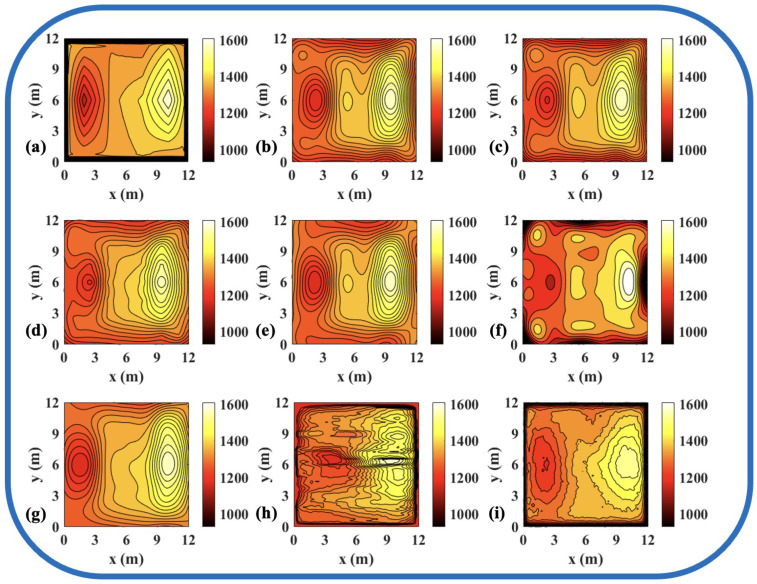
Reconstruction results of experimental high-low temperature field in 96 paths. (**a**) Discrete field. (**b**) LSM_IMQ. (**c**) LSM_IQ. (**d**) LSM_MK. (**e**) LSM_MQ. (**f**) LSM_RS. (**g**) LSM. (**h**) CNN. (**i**) ATTRN.

To validate the effectiveness of the ATTRN framework in engineering applications, we selected three discrete temperature fields for testing. The evaluation metrics $E_{\max}$ and $E_{\mathrm{rms}}$ are the same as those defined in Equations (14) and (15). The reconstruction errors are presented in Table 10 and Table 11, while the reconstructed temperature distributions are illustrated in Figure 7, Figure 8, Figure 9, Figure 10, Figure 11 and Figure 12.

The results demonstrate that, compared to the traditional LSM-based and CNN-based method, ATTRN achieves significant improvements in central, edge, and global error metrics. In real data sets of discrete fields in 54 and 96 paths, the global error of ATTRN is approximately 2.3%, significantly lower than the range of 4–7% observed in traditional methods and the CNN-based method. In addition, the edge error of ATTRN is approximately 3.4%, which remains markedly lower than the 5–9% range observed in traditional and CNN-based methods. Furthermore, with respect to central error, traditional LSM and CNN approaches exhibit values approximately 1% higher than ATTRN. Considering that ATTRN’s central error is only around 1.4%, this margin is relatively significant. In addition to lower average errors, ATTRN also demonstrates clear advantages in terms of $E_{\max}$. In the 54-path real data sets of discrete fields, ATTRN achieves the lowest central $E_{\max}$ at 71.63 °C, which is significantly lower than those of LSM-based approaches, ranging from 82.82 °C to 120.36 °C, and the CNN-based method, which reaches 126.27 °C. Similarly, for edge regions, ATTRN’s $E_{\max}$ is only 216.14 °C, whereas LSM-based approaches range from 226.15 °C to 427.65 °C, and the CNN-based method reaches 307.26 °C. A similar trend is observed in the 96-path configuration. ATTRN yields a central $E_{\max}$ of just 62.49 °C, while traditional methods and CNN report significantly higher values ranging from 96.06 °C to 190.89 °C. For edge regions, ATTRN once again achieves the lowest $E_{\max}$ at 192.23 °C, compared to 294.90 °C to 549.44 °C in the LSM-based and 209.87 °C in the CNN-based approach.

Thus, ATTRN consistently outperforms other comparison methods, exhibiting higher reconstruction accuracy under different sensor layouts and boundary conditions. This reinforces our belief that the acoustic information encoder-temperature field reconstruction decoder architecture that we designed effectively captures global temperature characteristics while suppressing noise interference, demonstrating strong temperature field reconstruction capabilities. By leveraging a Transformer-based AIE, the model enhances long-range dependency modeling, ensuring comprehensive encoding of acoustic path information. Meanwhile, the CNN-based TFRD further optimizes spatial details, resulting in more precise temperature distribution reconstruction. Consequently, this architecture significantly enhances the robustness and accuracy of temperature field reconstruction, making it well-suited for complex industrial measurement environments.

## 4. Conclusions

This study proposes a novel furnace temperature field reconstruction method based on an AIE-TFRD (ATTRN) model. By integrating Transformer and CNN architectures, the proposed method overcomes the limitations of traditional numerical approaches, such as cumulative error amplification, parameter dependency, and insufficient local detail capture. In the experimental phase, we constructed ATFRSD-54 and ATFRSD-96, which encompass various boundary conditions and sensor layouts, providing a comprehensive benchmark to evaluate the performance of ATTRN. The experimental results demonstrate that ATTRN achieves high reconstruction accuracy and robustness under different sensor configurations and boundary conditions. Notably, it maintains stable performance even in multi-heat-source and complex temperature field environments, exhibiting strong generalization capabilities. Compared to conventional LSM-based approaches, ATTRN significantly improves edge temperature feature recognition and global temperature distribution accuracy, offering a more reliable technological solution for furnace temperature monitoring and optimization control in power plants. Furthermore, validation using real-world data from the Chongqing Qineng Power Plant confirms that ATTRN effectively addresses the challenges of reconstructing highly dynamic and nonlinear temperature fields within boilers. This capability is critical for optimizing combustion control and ensuring operational safety. ATTRN demonstrates superior performance in terms of accuracy, noise resistance, and portability, making it adaptable to diverse operating conditions. It surpasses traditional iterative and non-iterative algorithms in temperature field reconstruction tasks. Given its feasibility and applicability in industrial scenarios, ATTRN can be further extended to more complex combustion systems and high-temperature environment monitoring.

In the future, it is worth noting that more rigorous physical modeling of wave propagation and refraction in non-uniform media remains a specialized and actively developing research domain [39,44,45,46,47]. Nevertheless, the present work primarily focuses on establishing a feasible temperature-field reconstruction framework for industrial furnaces and, therefore, adopts a simplified path-discretization approach. Future efforts will incorporate additional physical factors—particularly those arising from strong temperature gradients and three-dimensional acoustic ray-bending—by leveraging physics-informed neural networks (PINNs). By refining the acoustic ray-tracing algorithm, we aim to enhance reconstruction fidelity in the presence of pronounced refraction effects and curved propagation paths, thereby paving the way for complete 3D tomography of combustion and flow fields. Moreover, in scenarios involving highly non-uniform temperature distributions or significant velocity gradients, sound wave refraction may become the dominant factor affecting acoustic travel times. This highlights the potential benefits of integrating fully nonlinear acoustic models or curved-ray tracing algorithms into the framework. Finally, our long-term plan includes further validation and parameter refinement (e.g., cooling coefficients and path discretization schemes) using proprietary industrial datasets. These extensions will improve the physical rigor of the ATTRN framework and expand its applicability to real-world multi-physics acoustic thermometry tasks under challenging high-temperature environments.

## Figures and Tables

**Table 1:** Parameter ranges of water wall in the data generation code.

Parameter	Range/Values
Number of samples	6400 (per variant)
Background temperature	[1100, 1400]
Number of sources	{0, …, 8}
Source center $\left(A_{k},B_{k}\right)$	[3, 9] × [3, 9]
Source spreads $\left(C_{k},D_{k}\right)$	[3, 40]
Source amplitude $\left(E_{k}\right)$	[100, 600]
Cold-source probability	[0.3, 0.7]
Cooling iterations	{2, …, 6}
Cooling coefficient α	[0.03, 0.12]
Wall temperature	[600, 900]
Border width	[0.8, 1.6]

**Table 2:** Parameter ranges (no water wall) in the data generation code.

Parameter	Range/Values
Number of samples	6400 (per variant)
Background temperature	[1100, 1400]
Number of sources	{0, …, 10}
Source center $\left(A_{k},B_{k}\right)$	[0, 12] × [0, 12]
Source spreads $\left(C_{k},D_{k}\right)$	[3, 40]
Source amplitude $\left(E_{k}\right)$	[100, 600]
Cold-source probability	[0.3, 0.7]

**Table 3:** Reconstruction error on ATFRSD-54W test set.

Reconstruction Algorithm	Central Error		Edge Error		Global Error
	$E_{\max}$ (°C)	$E_{\mathrm{rms}}$	$E_{\max}$ (°C)	$E_{\mathrm{rms}}$	$E_{\mathrm{rms}}$
LSM	189.24	9.80%	-	-	-
LSM_IMQ	245.34	10.26%	257.46	7.35%	9.08%
LSM_IQ	266.29	10.90%	345.30	7.47%	9.54%
LSM_MK	234.33	9.79%	248.55	6.92%	8.62%
LSM_MQ	234.22	9.52%	351.84	10.47%	9.84%
LSM_RS	327.30	13.92%	626.83	14.09%	13.81%
CNN	1224.37	21.18%	1151.60	20.55%	20.95%
ATTRN	76.21	2.77%	181.39	4.78%	3.69%

**Table 4:** Reconstruction error on ATFRSD-54 test set.

Reconstruction Algorithm	Central Error		Edge Error		Global Error
	$E_{\max}$ (°C)	$E_{\mathrm{rms}}$	$E_{\max}$ (°C)	$E_{\mathrm{rms}}$	$E_{\mathrm{rms}}$
LSM	120.05	3.50%	-	-	-
LSM_IMQ	171.65	3.90%	297.73	6.04%	5.00%
LSM_IQ	191.45	4.48%	419.05	9.01%	6.86%
LSM_MK	163.35	3.45%	289.41	5.56%	4.55%
LSM_MQ	167.22	3.71%	280.93	5.01%	4.31%
LSM_RS	252.51	7.46%	702.44	16.90%	12.39%
CNN	1324.83	20.74%	1336.45	16.02%	18.83%
ATTRN	104.47	2.95%	132.28	2.96%	3.39%

**Table 5:** Reconstruction error on ATFRSD-96W test set.

Reconstruction Algorithm	Central Error		Edge Error		Global Error
	$E_{\max}$ (°C)	$E_{\mathrm{rms}}$	$E_{\max}$ (°C)	$E_{\mathrm{rms}}$	$E_{\mathrm{rms}}$
LSM	751.04	12.59%	-	-	-
LSM_IMQ	1062.15	13.93%	1257.21	24.04%	22.09%
LSM_IQ	1062.06	13.80%	1192.60	22.73%	21.51%
LSM_MK	991.25	10.03%	1015.39	20.12%	18.00%
LSM_MQ	1063.42	13.96%	1341.94	25.45%	22.64%
LSM_RS	1082.34	16.32%	1689.69	34.07%	28.06%
CNN	1227.39	15.10%	1170.17	20.60%	20.93%
ATTRN	68.72	2.55%	149.02	3.77%	3.09%

**Table 6:** Reconstruction error on ATFRSD-96 test set.

Reconstruction Algorithm	Central Error		Edge Error		Global Error
	$E_{\max}$ (°C)	$E_{\mathrm{rms}}$	$E_{\max}$ (°C)	$E_{\mathrm{rms}}$	$E_{\mathrm{rms}}$
LSM	205.69	5.86%	-	-	-
LSM_IMQ	305.04	6.44%	420.61	8.50%	7.35%
LSM_IQ	313.33	6.62%	481.48	9.89%	8.04%
LSM_MK	280.21	5.28%	361.08	6.65%	5.84%
LSM_MQ	301.47	6.37%	419.11	8.08%	7.12%
LSM_RS	387.40	9.76%	1037.23	23.55%	16.52%
CNN	1324.83	20.78%	1176.10	15.32%	18.60%
ATTRN	97.71	3.77%	115.64	2.71%	3.20%

**Table 7:** The 54-path model parameters.

54 Paths ATTRN Model	Parameter Size
Number of tokens, *N*	54
Embedded dimension, $D_{1}$	156
FFN Dimension in stage 1 Transformer encoder	312
Number of layers in stage 1 Transformer encoder	3
Number of heads in stage 1 Transformer encoder	4
Number of expanded tokens, $T_{\exp}$	256
Dimension of expanded token	32
FFN Dimension in stage 2 Transformer encoder	64
Number of layers in stage 2 Transformer encoder	1
Number of heads in stage 2 Transformer encoder	4
All ResBlock convolutional kernel size	3×3
All up convolution layer kernel size	4×4
Final convolution layer kernel size	3×3
Model parameters size	265.7 MB
Model FLOPs	0.1484 GFLOPs

**Table 8:** The 96-path model parameters.

96 Paths ATTRN Model	Parameter Size
Number of tokens, *N*	96
Embedded dimension, $D_{1}$	156
FFN Dimension in stage 1 Transformer encoder	312
Number of layers in stage 1 Transformer encoder	3
Number of heads in stage 1 Transformer encoder	4
Number of expanded tokens, $T_{\exp}$	256
Dimension of expanded token	32
FFN Dimension in stage 2 Transformer encoder	64
Number of layers in stage 2 Transformer encoder	1
Number of heads in stage 2 Transformer encoder	4
All ResBlock convolutional kernel size	3×3
All up convolution layer kernel size	4×4
Final convolution layer kernel size	3×3
Model parameters size	470.4 MB
Model FLOPs	0.2268 GFLOPs

**Table 9:** Burner model parameters.

Project	Parameter
Rated Power	330 MW
Burner Model	DG1114/18.5-II15
Number of Burners	24
Maximum Continuous Evaporation (B-MCR)	1114 t/h
Superheater Outlet Steam Pressure	18.5 MPa (gauge pressure)
Superheater Outlet Steam Temperature	543 °C
Reheated Steam Flow Rate	936.69 t/h
Reheater Inlet Steam Pressure	4.02 MPa (gauge pressure)
Reheater Outlet Steam Pressure	3.80 MPa (gauge pressure)
Reheater Inlet Steam Temperature	332 °C
Reheater Outlet Steam Temperature	543 °C
Economizer Inlet Feedwater Temperature	280.06 °C

**Table 10:** Reconstruction error of discrete fields in 54 paths.

Reconstruction Algorithm	Central Error		Edge Error		Global Error
	$E_{\max}$ (°C)	$E_{\mathrm{rms}}$	$E_{\max}$ (°C)	$E_{\mathrm{rms}}$	$E_{\mathrm{rms}}$
LSM	82.82	2.80%	-	-	-
LSM_IMQ	98.56	3.02%	278.11	7.51%	4.74%
LSM_IQ	97.18	2.88%	226.15	5.42%	4.10%
LSM_MK	110.81	3.26%	299.05	7.93%	5.05%
LSM_MQ	111.06	3.66%	427.65	9.01%	6.86%
LSM_RS	120.36	3.69%	312.48	8.63%	6.10%
CNN	126.27	2.11%	307.26	5.91%	4.06%
ATTRN	71.63	1.68%	216.14	3.63%	2.62%

**Table 11:** Reconstruction error of discrete fields in 96 paths.

Reconstruction Algorithm	Central Error		Edge Error		Global Error
	$E_{\max}$ (°C)	$E_{\mathrm{rms}}$	$E_{\max}$ (°C)	$E_{\mathrm{rms}}$	$E_{\mathrm{rms}}$
LSM	118.05	1.53%	-	-	-
LSM_IMQ	145.47	2.58%	335.23	5.27%	4.53%
LSM_IQ	141.36	2.43%	294.90	4.49%	3.86%
LSM_MK	96.06	1.92%	337.28	5.56%	4.77%
LSM_MQ	149.16	2.70%	428.04	6.34%	5.43%
LSM_RS	190.89	2.73%	549.44	5.95%	5.16%
CNN	148.81	2.63%	209.87	4.97%	3.72%
ATTRN	62.49	1.04%	192.23	3.35%	2.26%

## Data Availability

Data are contained within the article.
